# Huge Abdominal Desmoid Tumor Successfully Managed by Chemotherapy in a Young Patient With Familial Adenomatous Polyposis: A Case Report

**DOI:** 10.7759/cureus.107811

**Published:** 2026-04-27

**Authors:** Soundous Bennour, Farah Boutaagount, Meryem Maskrout, Rania Mokfi, Chaymae Senoussi, Ghizlane Rais

**Affiliations:** 1 Medical Oncology, Faculty of Medicine and Pharmacy of Agadir, Ibn Zohr University, Centre Hospitalier Universitaire Souss Massa, Agadir, MAR

**Keywords:** apc gene mutation, desmoid tumor, familial adenomatous polyposis, medical management, neoadjuvant chemotherapy

## Abstract

Familial adenomatous polyposis (FAP) is an inherited disorder characterized by the development of numerous colorectal polyps and an increased risk of extracolonic manifestations, notably desmoid tumors. Desmoid tumors are benign but locally aggressive fibrous neoplasms that do not metastasize but have a high tendency to recur. Due to their rarity, desmoid tumors often present with varied clinical features and nonspecific symptoms. We report the case of a 24-year-old man with no prior medical history who presented in March 2019 with melena, rectal bleeding, and digestive discomfort, without fever or systemic signs. Colonoscopy revealed polyposis, prompting a total colectomy with lymph node dissection in November 2019. Histopathology confirmed approximately 30 tubular adenomas with mild dysplasia; all lymph nodes were free of disease. In October 2020, the patient developed an obstructive syndrome managed by stoma creation, with successful intestinal continuity restoration in February 2021. In May 2021, abdominal swelling led to the discovery of a 17 cm ulcerated mass below the umbilicus. Imaging identified a large necrotic abdominopelvic mass and a smaller intraperitoneal mass. Histological and immunohistochemical analyses confirmed deep fibromatosis (desmoid tumor). The patient received vinblastine and methotrexate chemotherapy, resulting in significant tumor reduction. Subsequently, on September 1, 2023, he underwent extensive tumor resection with lymph node dissection. Pathology confirmed a 7 × 7 × 3.2 cm desmoid tumor with clear margins. This case highlights the critical need for vigilant monitoring in FAP patients, emphasizing early diagnosis and a multimodal treatment approach combining systemic therapy and surgery to effectively manage desmoid tumors.

## Introduction

Desmoid tumors, also known as desmoid-type fibromatosis or aggressive fibromatosis, are benign mesenchymal neoplasms characterized by monoclonal proliferation. They belong to the myofibroblastic fibromatosis family and are marked by aggressive local invasion of surrounding tissues, unpredictable growth patterns, and a high likelihood of recurrence, despite lacking metastatic potential [1]. Desmoid-type fibromatosis is an exceptionally rare neoplasm, accounting for approximately 3% of all soft-tissue tumors, with an estimated incidence of 2-4 cases per million individuals. Desmoid tumors may occur sporadically or in association with familial adenomatous polyposis (FAP) [2].

Sporadic desmoid tumor oncogenesis is typically linked to physiological and endocrine factors, including estrogen hormonal stimulation and pregnancy [3]. Trauma and prior surgical interventions are associated with approximately 25% of desmoid tumor cases [4]. Radical surgery, involving wide tumor resection, was initially considered the primary treatment approach for desmoid tumors; however, local recurrence rates following surgical resection ranged from 19% to 77% [5]. Consequently, additional therapies such as radiotherapy, chemotherapy, hormonal inhibitors, and non-hormonal anti-inflammatory agents have been introduced, either as adjuncts to surgery or as first-line treatments in more modern, conservative management strategies [6].

Desmoid tumors are characterized by an unpredictable clinical course, with periods of stability, progression, or spontaneous regression, making their management particularly challenging [7]. Despite these advances, uncertainty remains regarding the optimal management strategy, especially in patients with FAP, where disease behavior may be more aggressive and therapeutic decisions more complex. In this context, reporting individual cases remains important to illustrate clinical challenges and treatment responses.

We report an exceptional case of a large desmoid tumor in a young patient with FAP, showing a good response to neoadjuvant chemotherapy and successfully managed with a multimodal approach.

## Case presentation

**Figure 1:**
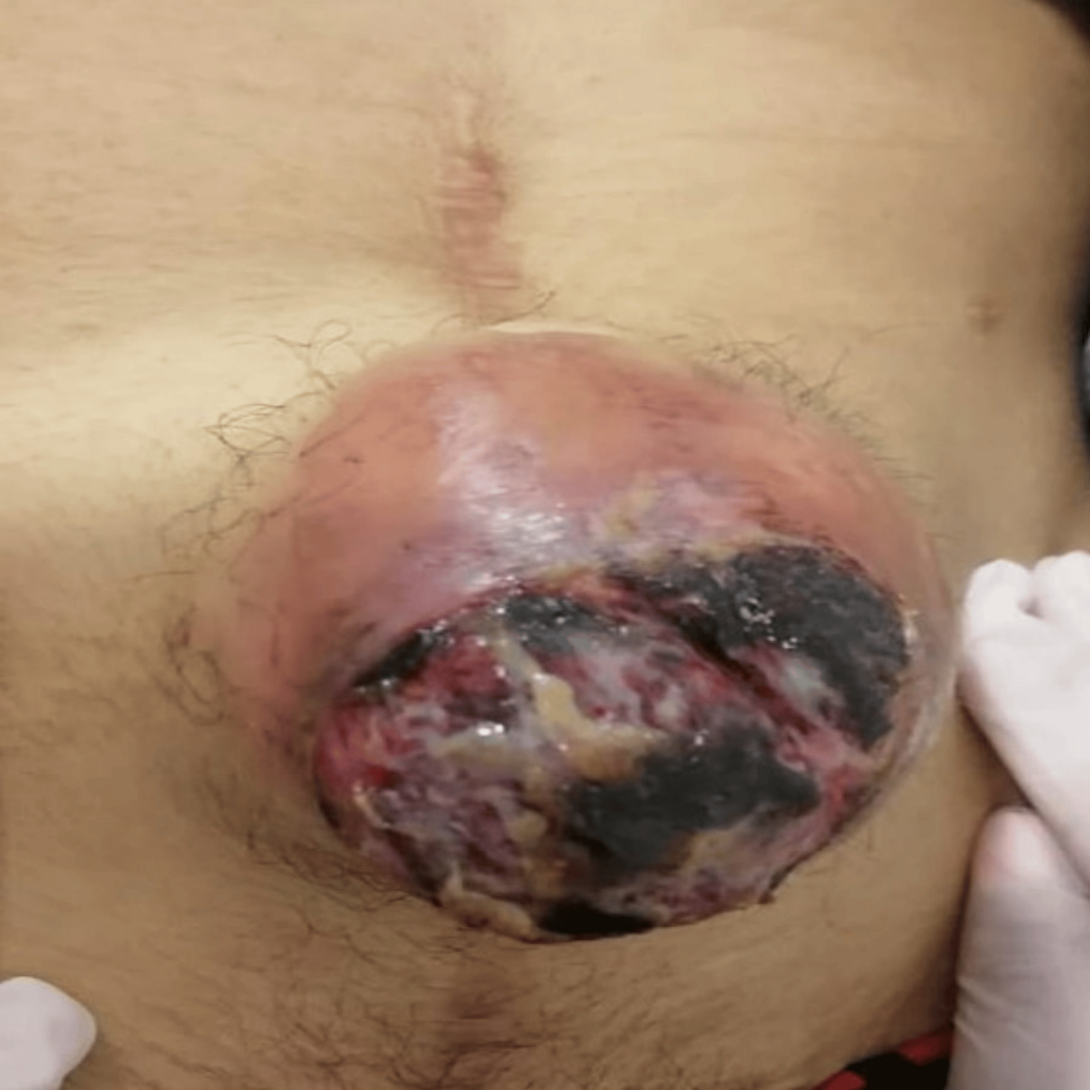
Clinical photograph showing a large, irregularly shaped ulcerated mass measuring 17 cm in the greatest diameter in the periumbilical region.

Mr. E.M., a 24-year-old male patient with no significant past medical history, presented in March 2019 with episodes of melena, rectal bleeding, and digestive issues, accompanied by general malaise without fever. A colonoscopy revealed polyposis, leading to a total colectomy with lymph node dissection in November 2019. Histopathological analysis identified approximately 30 simple tubular adenomas with mild dysplasia, measuring between 0.5 and 3 cm, with no involvement of the 31 lymph nodes examined. FAP was diagnosed based on clinical findings and family history. The patient had a suggestive familial background of colorectal polyposis, and colonoscopy demonstrated multiple colorectal adenomatous polyps consistent with FAP. The patient was closely monitored after surgery. In October 2020, he developed an obstructive syndrome, which was managed with stoma creation, followed by restoration of intestinal continuity in February 2021. After two months, a progressively enlarging mass was observed in the periumbilical region. Clinical examination revealed a 17 cm ulcerated mass in the periumbilical area (Figure 1), with no other associated symptoms.

**Figure 2:**
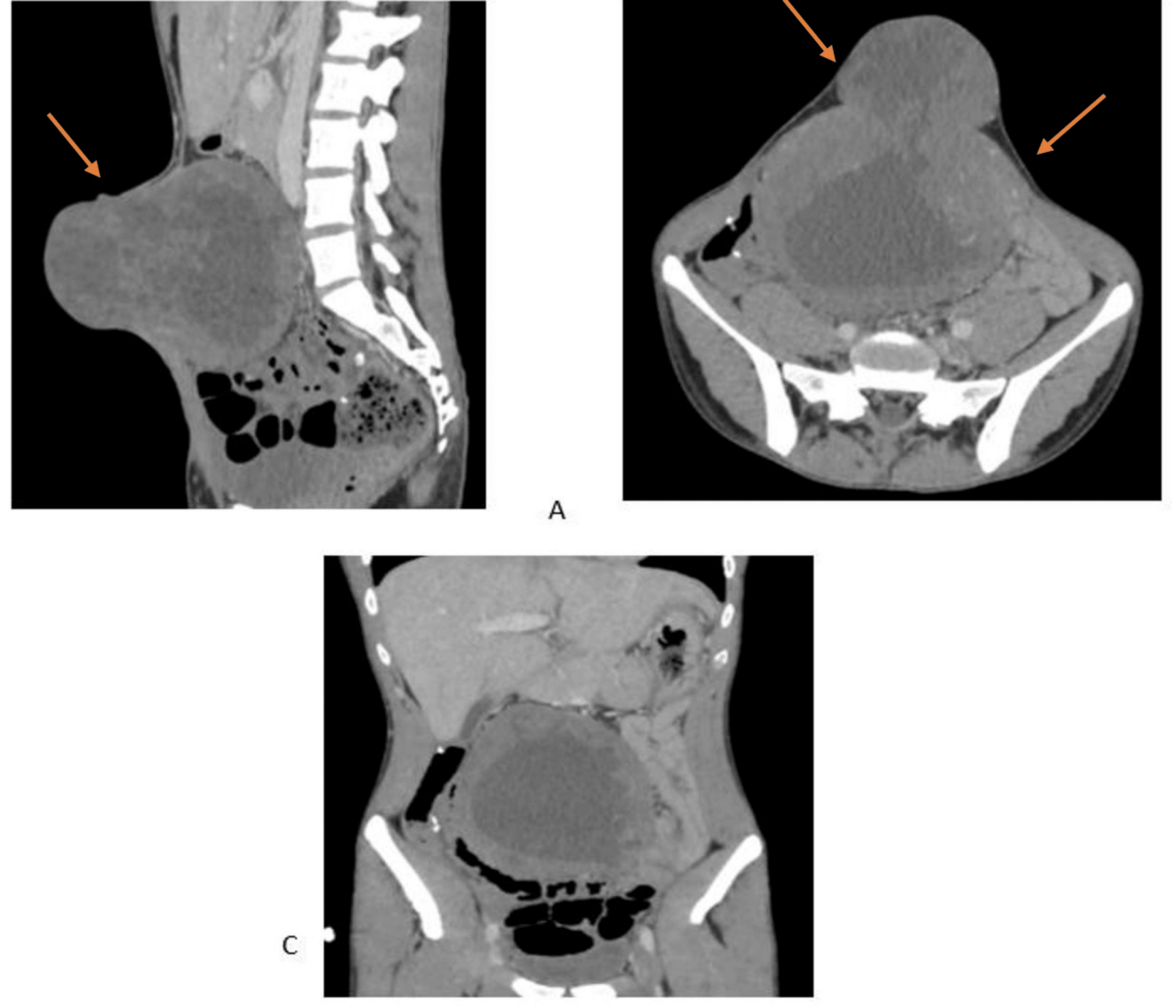
Coronal and sagittal views showing two peritoneal masses. The arrows in A and B indicate a large, extensively necrotic median abdominopelvic mass (158 × 132 × 123 mm) extending to the abdominal wall and herniating through the umbilicus, as well as a smaller intraperitoneal mass on the left (29 × 19 × 49 mm).

CT imaging identified two peritoneal masses: a large, extensively necrotic median abdominopelvic mass (158 × 132 × 123 mm) extending to the abdominal wall and herniating through the umbilicus, and a smaller intraperitoneal mass on the left (29 × 19 × 49 mm) (Figure 2).

**Figure 3:**
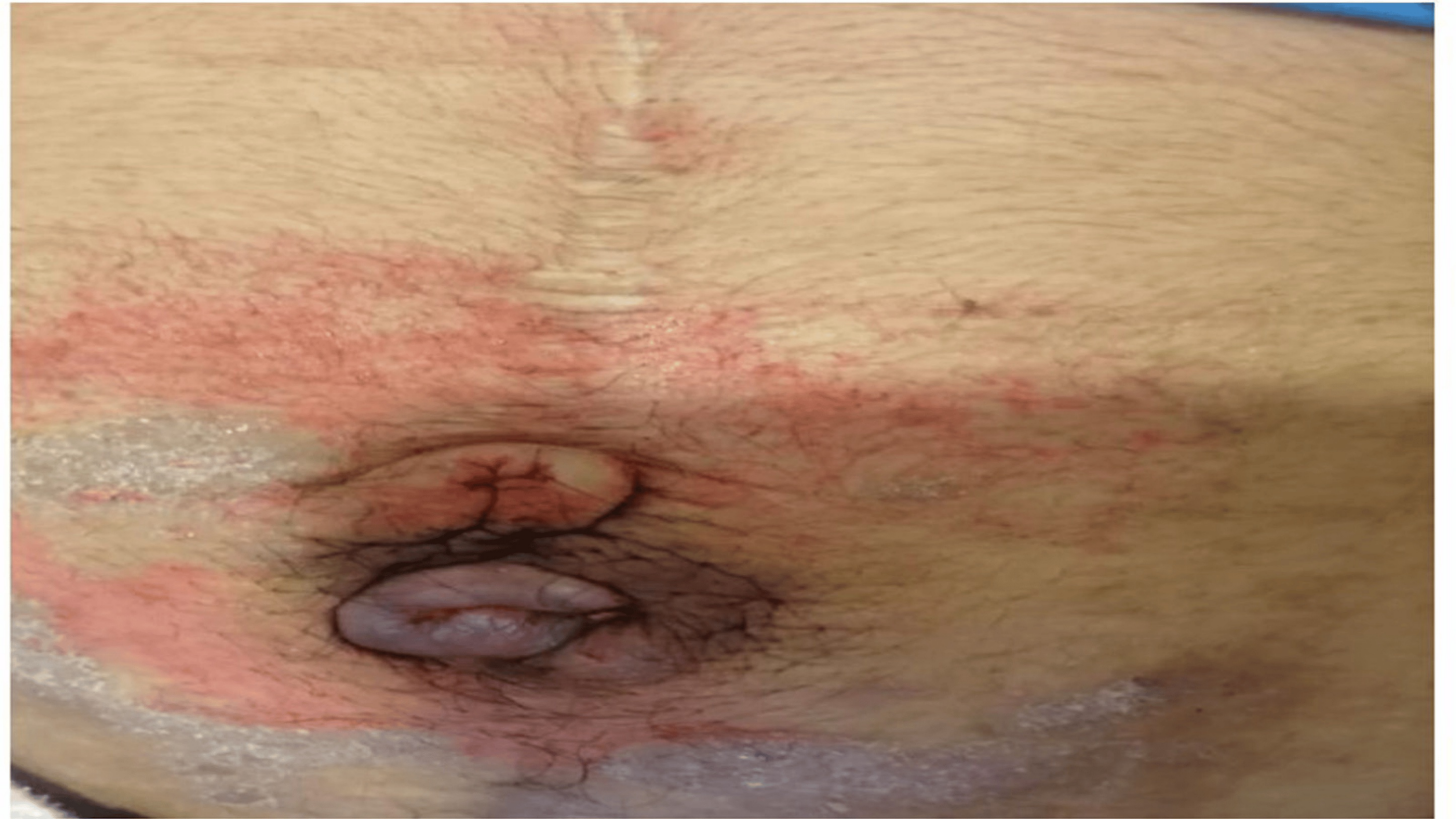
Clinical image showing clinical response to neoadjuvant chemotherapy.

**Figure 4:**
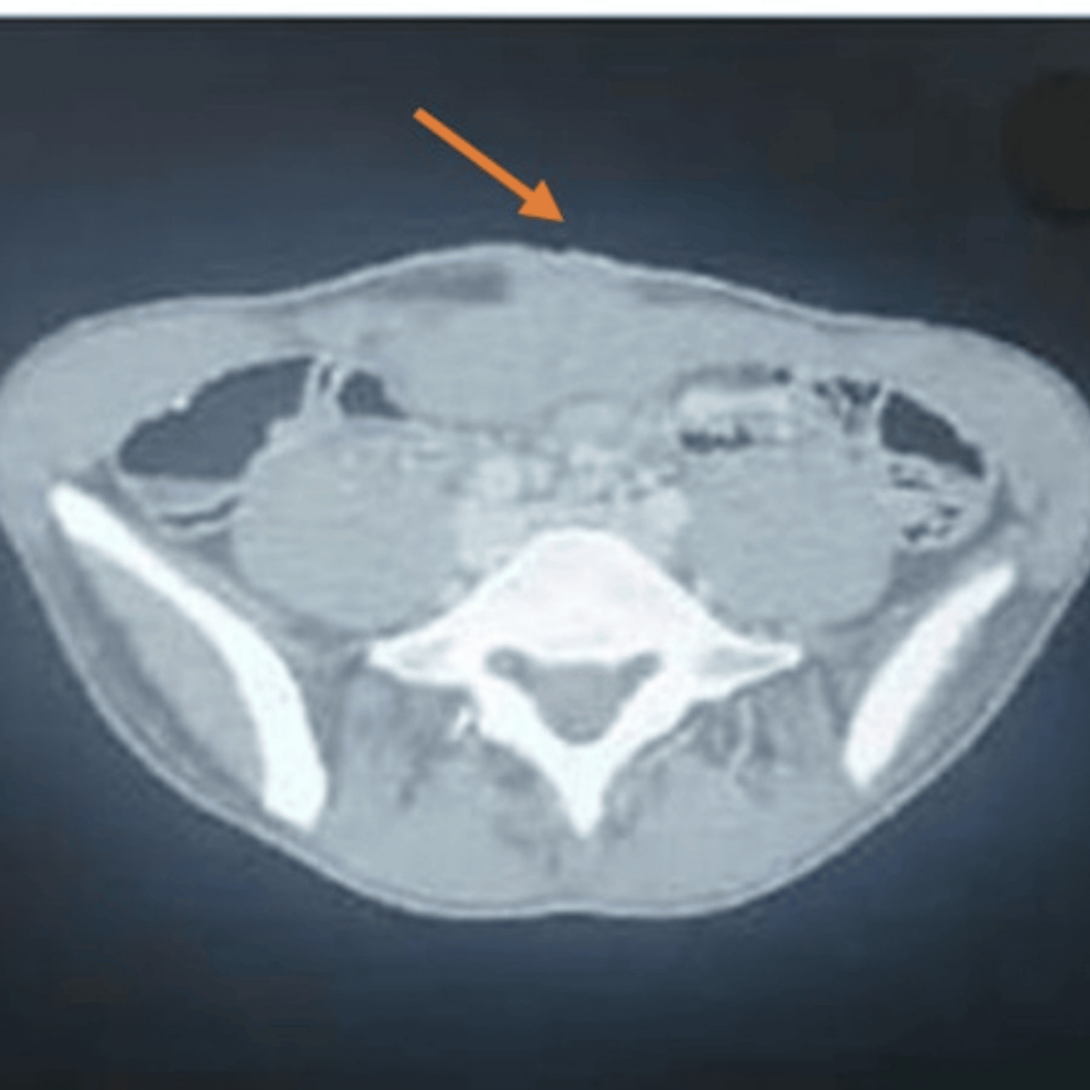
CT scan image demonstrating significant reduction in tumor size to 70 × 33 × 68 mm, as indicated by the arrow.

A Tru-Cut needle biopsy was performed to obtain histological confirmation. Microscopic analysis revealed a myxoid tumor with spindle cells, while immunohistochemistry showed moderate and heterogeneous positivity for anti-AML antibodies, strong positivity for anti-beta-catenin antibodies, and negativity for anti-desmin and anti-S100 antibodies. These findings were consistent with a desmoid tumor (deep fibromatosis). The case was discussed in a multidisciplinary team meeting (MDT), and the decision was made to initiate neoadjuvant chemotherapy with methotrexate and vinblastine. The patient received a chemotherapy regimen consisting of methotrexate (30 mg/m²) and vinblastine (6 mg/m²), administered every 14 days, with good tolerance and no significant adverse events reported, resulting in significant clinical (Figure 3) and radiological improvement, with the masses reducing to 70 × 33 × 68 mm, according to radiologic response criteria (Figure 4).

**Figure 5:**
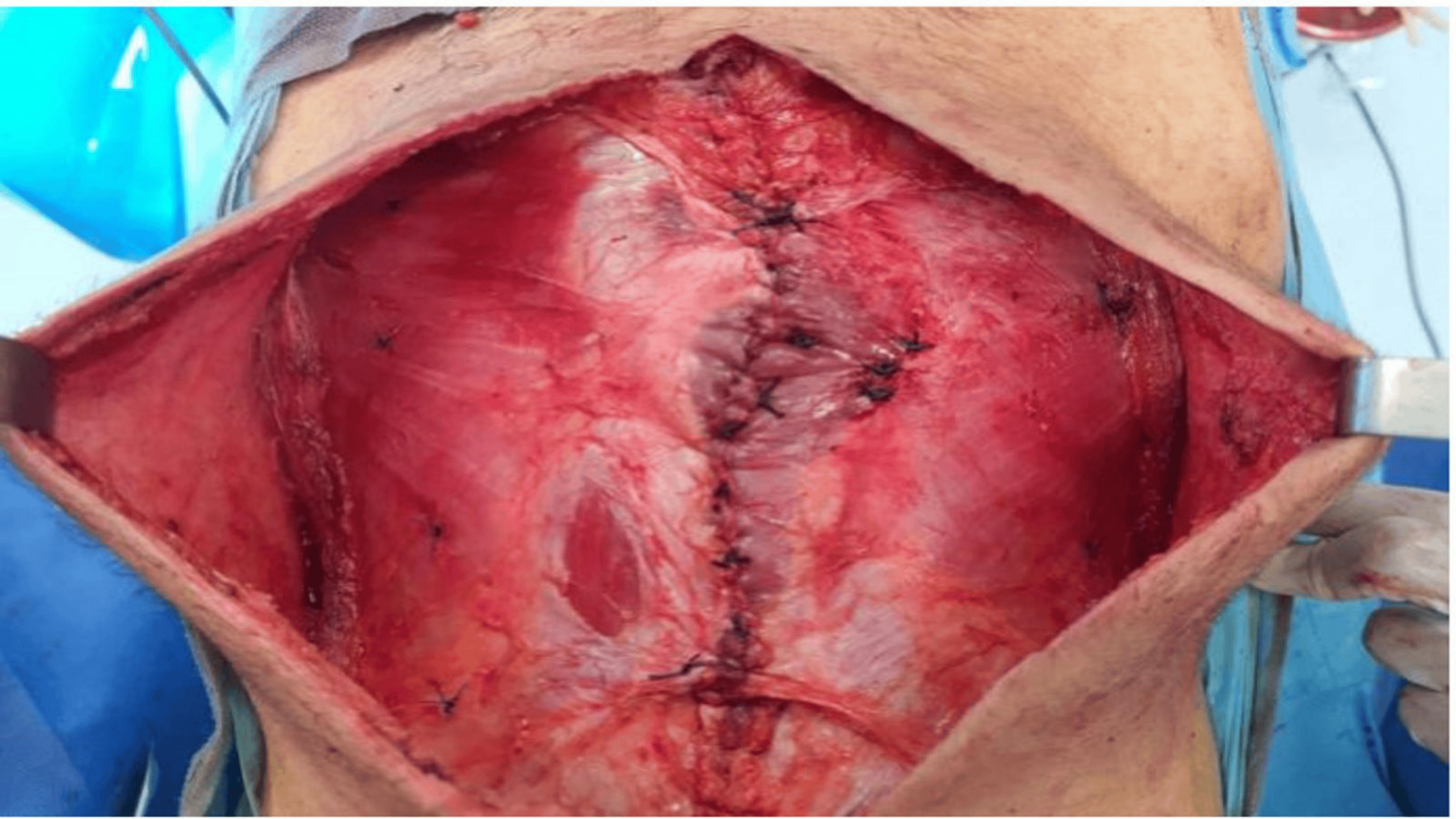
Extensive parietal resection with resection of peritoneal nodules.

Given this clinical and radiological improvement, the patient was reassessed in the MDT, and surgery was decided. The patient underwent extensive parietal resection with lymph node dissection (Figure 5).

Pathological analysis confirmed a desmoid tumor measuring 7 × 7 × 3.2 cm with clear surgical margins, which confirmed an R0 resection with all surgical margins free of tumor. The case was reassessed in an MDT, and adjuvant chemotherapy was decided to complete a total of 18 cycles. The patient received the chemotherapy, and follow-up evaluations were performed, including an MRI every six months, abdominal ultrasound every three months, and an annual fibroscopy and colonoscopy. The patient remained free of recurrence 16 months after completing treatment.

## Discussion

Desmoid tumors, also referred to as deep fibromatosis, are rare soft-tissue neoplasms that arise from musculoaponeurotic structures. They account for fewer than 0.03% of all tumors and approximately 3.5% of fibrous tumors [1]. Desmoid tumors can arise in multiple anatomical sites, including the abdominal wall, limbs, girdles, head and neck, breast, pelvis, retroperitoneum, and mesentery [8]. The annual incidence is estimated at 2-4 cases per million individuals, with a strong association with FAP, in which the lifetime risk of developing a desmoid tumor is approximately 10% to 20% [2].

The pathogenesis of desmoid tumors involves genetic predisposition, hormonal factors, and mechanical stress. About 85% of sporadic desmoid tumors harbor mutations in the *CTNNB1* gene, particularly the 41A and 45F mutations, which have been linked to higher recurrence rates [5]. In FAP-associated cases, *APC* gene mutations result in β-catenin overexpression, leading to dysregulated fibroblastic proliferation [6]. FAP is associated with the development of desmoid tumors in approximately 10-20% of affected individuals [9]. These FAP-related desmoids represent between 5% and 15% of all desmoid tumors [10]. In patients with FAP, desmoid tumors represent a major cause of morbidity and are most frequently located intra- abdominally or in the abdominal wall [11].

FAP is caused by mutations in the *APC* gene, located on chromosome 5q21-q22. In most cases, desmoid tumors in FAP patients are found within the abdominal cavity or along the abdominal wall [12]. The term Gardner syndrome is used when individuals with FAP also develop extraintestinal manifestations, such as desmoid tumors. In some patients, a desmoid tumor may be the only clinical sign of an underlying *APC* gene mutation [13,14]. Additional risk factors include prior surgical interventions, trauma, and pregnancy-related hormonal changes, particularly elevated estrogen levels [3]. Most individuals diagnosed with a desmoid tumor are between 15 and 60 years old. These tumors occur slightly more often in females than in males [15], with no notable racial or ethnic preference.

Desmoid tumors typically present as slow-growing, painless masses. The natural history of desmoid tumors is unpredictable. These lesions are often found incidentally during imaging studies conducted for other reasons. Typically, they exhibit slow growth and are frequently diagnosed at an advanced stage, after causing local infiltrative and destructive effects that are proportional to their size and location. As a result, symptoms correlate with the affected area, potentially leading to intestinal, vascular, neurologic, or genitourinary obstruction. Clinical manifestations can include pain, functional impairment, bowel obstruction, and, in severe cases, perforation that requires extensive bowel resection [7]. The symptoms vary depending on the location. Abdominal wall and intra-abdominal desmoid tumors, as seen in this case, can cause bowel obstruction, ischemia, or ureteral compression [4]. While they lack metastatic potential, their aggressive local invasion results in significant morbidity, requiring careful management [16]. The diagnosis of desmoid tumors relies on imaging and histopathology.

Among available modern imaging modalities, MRI is preferred over CT for its superior soft-tissue contrast and ability to distinguish desmoid tumors from malignant soft-tissue tumors [2]. On contrast-enhanced CT scans, these tumors typically exhibit attenuation equal to or greater than that of muscle tissue. Their margins may appear well-defined or ill-defined, depending on the extent of infiltration into the surrounding structures [17]. On MRI scans, desmoid tumors exhibit low signal intensity compared to muscle on T1-weighted images and variable signal intensity on T2-weighted images [17]. MRI plays a crucial role in assessing the behavior of desmoid tumors. A bright signal on T2-weighted images reflects increased water content, which has been linked to accelerated tumor growth. This correlation suggests that tumors with higher T2 signal intensity may exhibit greater biological activity and a higher likelihood of progression [18]. Desmoid tumors do not exhibit distinct imaging characteristics that unequivocally differentiate them from other solid masses. However, their diagnosis should be considered in patients presenting with an abdominal mass, particularly those with a history of abdominal surgery or trauma, or an association with FAP [17].

The histological diagnosis of desmoid tumors is primarily achieved through image-guided core needle biopsy, using 14- to 16-gauge needles. This method is preferred for its accuracy and lower risk compared to surgical biopsy, which is generally avoided due to potential complications and tumor progression [17,19]. A definitive diagnosis relies on histopathological examination, which reveals myxoid spindle cell proliferation. Histologically, desmoid tumors are characterized by a monoclonal [15] fibroblastic proliferation appearing as small bundles of spindle cells in an abundant fibrous stroma.

The fibroblasts have a propensity to concentrate at the periphery of the lesion, and the cellularity is low. The infiltrative connective tissue process may resemble that of a low-grade fibrosarcoma, but the cells lack nuclear and cytoplasmic features of malignancy. There are usually few mitotic figures, and necrosis is absent. Histologically, sporadic and familial adenomatous polyposis-associated desmoids are indistinguishable. with strong β-catenin positivity and absence of desmin and S100 in immunohistochemistry, distinguishing them from fibrosarcomas [5]. Molecular studies confirming *CTNNB1* or APC mutations can further aid in diagnosis [7]. In our case, the diagnosis was established based on the characterization of two peritoneal masses on CT, and immunohistochemical analysis supported the diagnosis, favoring desmoid tumor.

The literature indicates that in patients with familial polyposis who have undergone subtotal colectomy or coloproctectomy, the development of a mesenteric mass, particularly near the mesentery of the ileal reservoir, is highly suggestive of a desmoid tumor [20]. Desmoid tumors can follow an unpredictable clinical course, with some tumors undergoing regression in the absence of therapy. For many patients, especially those with asymptomatic or minimally symptomatic tumors, an initial strategy of active surveillance is the preferred approach. In some cases, spontaneous regression of desmoid tumors has been observed. One study found that 10% of desmoid tumors resolved on their own, while 50% remained stable after diagnosis, and 10% exhibited rapid progression. Spontaneous regression has been mainly reported in postmenopausal women or those undergoing oophorectomy, further supporting the relationship between desmoid tumors and estrogen levels [21,22].

However, if a desmoid tumor progresses on serial imaging or is associated with significant symptoms that are difficult to manage, an MDT should determine the best individualized approach. Although surgery and radiotherapy were traditionally used in the management of desmoid tumors, their role has become selective due to high recurrence rates and the risk of significant morbidity, particularly in patients with intra-abdominal or mesenteric disease. Therefore, systemic therapy is often preferred when an intervention is required, especially in patients with FAP or intra abdominal/mesenteric tumors. In a systematic review of the surgical management of abdominal desmoid tumors, the authors analyzed surgical strategies and outcomes, reporting high recurrence rates and significant postoperative morbidity, particularly in intra-abdominal and mesenteric disease [23].

Chemotherapy is an option for unresectable desmoid tumors or when surgery carries high morbidity. The management of desmoid tumors is highly individualized, taking into account tumor behavior, symptoms, comorbidities, and patient preferences. Pegylated liposomal doxorubicin (PLD) is often the preferred first-line cytotoxic agent in patients requiring chemotherapy, mainly due to its more favorable safety profile compared to conventional doxorubicin-based combination regimens. Although combination therapies such as doxorubicin plus dacarbazine may yield higher response rates, they are associated with significantly increased toxicity. Hematologic and cardiac adverse effects are more common, and there is no clear evidence that these regimens improve progression-free survival. For patients who cannot tolerate or wish to avoid cytotoxic chemotherapy, nirogacestat, a gamma secretase inhibitor, serves as a reasonable first-line alternative, provided the patient can take oral medication without issue. Cytotoxic chemotherapy has demonstrated response rates ranging from 17% to 100%, with a median of about 50% [24]. Importantly, patients may experience functional and symptomatic improvement, including pain relief, as early as four weeks after initiating treatment [25,26], even before any radiological evidence of tumor shrinkage becomes apparent. However, the optimal duration of treatment has yet to be clearly defined and remains under discussion. Anthracycline monotherapy, especially with PLD, is commonly used for most patients requiring chemotherapy because it tends to produce fewer side effects compared to unencapsulated doxorubicin. PLD is associated with lower rates of myelosuppression, febrile neutropenia, alopecia, and cardiotoxicity [27]. In some series, anthracyclines have demonstrated response rates exceeding 75% [28]. In specific clinical scenarios where rapid tumor control is essential, such as in cases of aggressive, symptomatic progression that are unresponsive to local or systemic therapy, combination chemotherapy may be considered. This approach is reserved for selected patients due to its higher toxicity. For example, in one of the largest case series, a regimen of doxorubicin (60 to 90 mg/m²) combined with dacarbazine (750 to 1,000 mg/m², continuously infused over 72 hours) administered over a median of five cycles produced an objective response in six of nine evaluable patients, including two complete and four partial responses [25]. Nevertheless, this regimen was also associated with notable adverse effects, including cardiac toxicity, myelosuppression, mucositis, and nausea. An alternative approach involves the use of low-dose methotrexate in combination with a vinca alkaloid such as vinblastine or vinorelbine. Clinical studies have reported disease control rates, defined as tumor regression or stable disease, ranging from 70% to 100% [29-32]. Despite the frequent use of this combination, there is no compelling evidence that it is more effective than either agent used alone. Among vinca alkaloids, vinorelbine is sometimes preferred because it carries a lower risk of neurotoxicity compared to vinblastine [32].

Other systemic therapies, including nonsteroidal anti-inflammatory drugs, hormonal therapy, and tyrosine kinase inhibitors, have demonstrated efficacy in unresectable cases [5,19]. In our case, neoadjuvant chemotherapy with vinblastine and methotrexate was administered to shrink the tumor before surgical intervention, yielding a positive clinical response. which has proven effective in cases with extensive disease [6].

The prognosis of desmoid tumors is influenced by factors such as patient age, tumor location, size, and recurrence history. Younger individuals and those with larger tumors, particularly in the extremities, have a higher risk of recurrence. In contrast, abdominal wall tumors are associated with more favorable outcomes. A predictive model incorporating these variables has been developed to estimate recurrence risk and guide treatment decisions [33]. Due to the high recurrence rate of desmoid tumors, long-term surveillance is crucial. Standard follow-up includes MRI every six months, abdominal ultrasound every three months, and annual endoscopic assessments such as fibroscopy and colonoscopy. Additionally, monitoring β-catenin expression and conducting genetic profiling may aid in assessing recurrence risk and guiding personalized management [34].

This case highlights the diagnostic and management challenges of desmoid tumors in patients with FAP. focusing on their diagnosis, treatment, prognosis, and post-treatment surveillance. Given their unpredictable behavior, high recurrence rates, and the challenges in therapeutic decision-making, this study aims to consolidate the latest evidence-based approaches. By highlighting key diagnostic tools, treatment strategies, and prognostic factors, it seeks to guide clinicians in optimizing patient management. Additionally, the discussion on molecular markers and emerging therapies offers valuable insights into future directions for personalized treatment.

## Conclusions

This case underscores the necessity of a multidisciplinary, personalized approach in managing desmoid tumors, particularly in FAP-associated cases. The combination of neoadjuvant chemotherapy, surgical resection, and adjuvant therapy highlights the efficacy of multimodal treatment strategies. Given the unpredictable nature of desmoid tumors, long-term surveillance remains essential to detect recurrences early and optimize patient outcomes. Further research is needed to refine treatment algorithms and identify reliable biomarkers predictive of tumor behavior.
